# Impact of COVID-19 on Academic and Psychological Aspects in Students of Medicine: A Cross-Sectional Study

**DOI:** 10.7759/cureus.48259

**Published:** 2023-11-04

**Authors:** Riya Singla, Pratik Chatterjee, Prasanna Mithra

**Affiliations:** 1 Medicine and Surgery, Kasturba Medical College of Mangalore, Mangalore, IND; 2 Physiology, Kasturba Medical College of Mangalore, Mangalore, IND; 3 Community Medicine, Kasturba Medical College of Mangalore, Mangalore, IND

**Keywords:** challenges, stress, anxiety, lockdown, online education, pandemic, medical education

## Abstract

Background

The COVID-19 pandemic has caused a devastating disruption in medical education worldwide. The influence on training, mainly for undergraduate MBBS pupils, has been noteworthy, especially the significant and unexpected shift to online learning.

Materials and methods

We performed a two-month, cross-sectional study from June to August 2022 at Kasturba Medical College in Mangalore, India. The study participants were second- and third-year MBBS students at Kasturba Medical College. The sample size was 319. We collected data using a pre-structured, validated, printed questionnaire, then coded and entered the data into SPSS Statistics for Windows, version 25.0, for interpretation.

Results

A total of 319 people took part in the study. Of these, 60.2% were females, 39.4% were males, 71.8% were from the batch of 2019-2020, and the remaining 28.2% were from the batch of 2018-2019. Among the participants, 90 students suffered from COVID-19, including 72.7% (n = 65) from the second year. Twenty-four percent of the population did not contract COVID-19, including 88 from the second year. A total of 153 participants were unsure whether they were infected with COVID-19, including 57.5% from the second year and 42.5% from the third year. The study group's p-value of <0.0001 is statistically significant. On multivariate analysis, 54.5% of the study participants said the pandemic caused a significant disruption to their medical education. A majority of students (51.7%) agreed that the pandemic hampered practical/clinical work; 42.9% of participants somewhat disagreed that the pandemic hampered their interest in pursuing medical education in the future; and 21.9% of students said the pandemic hindered their interest in further studying medicine. Regardless of gender, a majority of the participants (78.1%) felt that online education negatively affected their time management skills and ability to cover the syllabus. Nearly half (46.4%) reported proficiency in using electronic devices. The medical students encountered multiple challenges: approximately 88% indicated that anxiety over the possibility of contracting the disease led to changes in personal behavior and interactions with family and friends. The study also revealed that 71% exhibited anxiety traits, and 11% displayed depressive symptoms, which may have been pre-existing. Furthermore, 77.1% of the participants experienced poor sleep quality, which, according to another study, is a significant predictor of depression and anxiety during COVID-19. Our findings show a significant correlation between undergoing COVID-19 testing and increased anxiety and stress levels among students, most of whom were infected during the pandemic's second wave in India.

Conclusion

The study has shed light on the effect of the COVID-19 pandemic on MBBS scholars and the students' reactions to this unparalleled situation. This aligns with Sustainable Development Goal 3, which focuses on "good health and well-being." The medical community has been significantly impacted by the pandemic due to its frontline position, and medical students' struggles to thrive academically. The knowledge gained from this study will assist facilitators and students of the medical fraternity in carrying out effective teaching modalities during this pandemic and any future outbreaks.

## Introduction

In 2019, the novel COVID-19 began and quickly evolved into a pandemic that wreaked havoc worldwide. As circumstances deteriorated, lockdowns around the globe led to social distancing. They culminated in the shuttering of educational institutions [1], causing an unparalleled interruption to the competency-based medical education (CBME) process and healthcare systems [2]. The impact on training, mainly for undergraduate MBBS pupils, has been noteworthy, especially with a pronounced and sudden shift to online learning. MBBS students are now exposed to online modules with audiovisual lessons and online assessments, potentially complicating their transition from student to medical practitioner. Medical students faced a lot of challenges, including fear of contracting the virus during training and transmitting it to the community. Students stayed home, abiding by social distancing guidelines, and attended online lectures, ensuring the continuity of the medical education process [3]. The amalgamation of condensed exposure to clinical meetings with deferral or annulment of add-ons and electives had perceptible effects on medical education. The efficacy of these newer methods in delivering holistic training is still questionable [4].

## Materials and methods

Study setting

We performed the study at Kasturba Medical College (KMC), Mangalore, at the Manipal Academy of Higher Education in Manipal, India, in the Dakshina Kannada district. 

Study design

This was a cross-sectional study [2].

Study population

We conducted this research among second- and third-year MBBS students at Kasturba Medical College, Mangalore, at the Manipal Academy of Higher Education in Manipal, India.

Study duration

The study lasted two months, from June to August 2022.

Sample size

We anticipated that 74.7% of medical students would report that COVID-19 influenced their education [5], with an absolute precision of 5% and a 95% confidence interval. Accounting for a 10% non-response rate, the total sample size required came to 319. This was calculated using a z-score of 1.96 for the 95% confidence interval, a proportion (p) of 0.747, the complement of the proportion (q) of 0.253, and a margin of error (d) of 5%, as per the formula n = (z^2)pq/d^2.

Sampling strategy

We used a convenience sampling (non-random) technique.

Inclusion criteria

We included medical students in their second and third years at Kasturba Medical College, Mangalore, at the Manipal Academy of Higher Education in Karnataka, India, who consented and were willing to participate in the study.

Exclusion criteria

We excluded students unwilling to participate in the study and students with psychiatric illnesses or on medications.

Tools for data collection

We gathered data through a pre-structured and pre-validated questionnaire, which was distributed electronically via Google Forms [6] and also available in printed format [2]. The questionnaire had two parts. The first part contained details of participants like age, gender, and address [2]. The second part of the questionnaire consisted of questions about the impact of COVID-19 on participants' daily routines, the challenges they faced during online classes, and their satisfaction with online training [6].

Data collection

We obtained clearance from the Institutional Ethics Committee (IEC) and permission from the dean of KMC Mangalore and the other required authorities before conducting this study. We visited the medical students in their classes on a pre-informed date and explained the study's objectives, following the necessary COVID-19 behavior(s). We provided participants with an information sheet and gathered their consent using a Google Form, which was distributed via the WhatsApp platform. Once students provided their consent, they were granted access to the questionnaire on the same Google Form. For those who preferred to participate offline, we offered a printed version of the questionnaire.

Data analysis

After collecting the data, we coded it and entered it into the IBM SPSS Statistics for Windows, version 25.0 (IBM Corp., Armonk, NY, USA) [2]. We stated the outcomes as parts by means of suitable tables and figures and used a Chi-square test, taking p ≤0.05 as statistically significant.

## Results

A total of 319 participants participated in this study, among whom 60.2% were females and 39.4% were males. The mean age was 20.30 (±0.808) years. Among the study participants, 71.8% were from the batch of 2019-2020, and the remaining 28.2% were from the batch of 2018-2019; 62.7% of the students resided in hostels, 15% were paying guests, and 21.6% were residents (Table 1).

**Table 1:** Characteristics of study participants (n=319)

Variables		n (%)
Gender		
	Male	127 (39.4%)
	Female	192 (60.2%)
Age range (years)		
	19–20	199 (62.3%)
	21–22	120 (37.6%)
Year of study		
	Second	229 (71.8%)
	Third	90 (28.2%)
Place of study		
	Hostel	200 (62.7%)
	Residents	69 (21.6%)
	Paying guest	50 (15.7%)

Table 2 depicts the characteristics of the study participants. Among the participants we recruited, 90 suffered from COVID-19, and 72.7% of those (n = 65) were in their second year. Twenty-four percent of the population did not contract COVID-19; 88 of these students were in their second year. A total of 153 participants were unsure whether they had been infected with COVID-19; 57.5% of these were second-year students, and 42.5% were third-year students. The p-value among the study group, <0.0001, is statistically significant (Table 2).

**Table 2:** Educational impact across the study group (n=319).

Serial no.	Statement	Strongly agree	Somewhat agree	Neither	Somewhat disagree	Strongly disagree
1.	My medical education has been significantly disrupted by the pandemic.	110 (34.5%)	174 (54.5%)	30 (9.2%)	2 (0.6%)	3 (0.9%)
2.	I believe my college is providing everything the best they can during this pandemic to adjust to students’ expectation(s)/issue(s).	142 (44.5%)	138 (43.5%)	27 (8.5%)	5 (1.6%)	7 (2.2%)
3.	I believe practical/clinical work has been hampered due to the pandemic.	132 (41.4%)	165 (51.7%)	18 (5.6%)	2 (0.6%)	2 (0.6%)
4.	I believe online classes have hampered interest in pursuing medical education in the future.	22 (6.9%)	70 (21.9%)	10 (3.1%)	137 (42.9%)	80 (25.1%)
5.	The quality of internet services affected efficacy in delivery of the content in class.	57 (17.9%)	32 (10.0%)	7 (2.2%)	150 (47.0%)	73 (22.9%)
6.	Lack of proficiency in using various electronic devices impacted effective learning.	30 (9.4%)	64 (20.1%)	2 (0.6%)	75 (23.5%)	148 (46.5%)
7	I experienced more distractions and less focus during online classes.	257 (80.6%)	39 (12.2%)	5 (1.6%)	10 (3.1%)	8 (2.5%)
8.	I felt online classes were less interactive than face-to-face classes.	288 (90.3%)	12 (3.8%)	2 (0.6%)	8 (2.5%)	9 (2.8%)
9.	I felt the online mode of education has affected time management in regard to covering the syllabus in time.	249 (78.1%)	29 (9.1%)	1 (0.3%)	23 (7.2%)	17 (5.3%)

Out of the study participants, 54.5% acknowledged the pandemic had significantly disrupted medical education; 44.5% of the students strongly agreed, and 43.5% somewhat agreed that their college adjusted to student's needs during the pandemic the best they could. A majority of students (51.7%) said the pandemic had hampered practical/clinical work; 42.9% somewhat disagreed that the pandemic had hampered interest in pursuing medical education in the future; and 21.9% said the pandemic hindered their interest in studying medicine. When asked whether online education had hindered their ability to manage time adequately and cover the syllabus, 78.1% said it had, irrespective of gender. However, the percentage of female participants strongly agreeing to this question (57.5%) was significantly higher than the percentage of male participants, with p < 0.046. A majority of the participants (46.4%) reported they were proficient in using electronic devices. Around 150 participants (47%) reported they had a good internet connection, while 27.9% of participants had a weak internet connection (Table 3).

**Table 3:** Health impact.

Serial no.	Statement	Strongly agree	Somewhat agree	Neither	Somewhat Disagree	Strongly Disagree
1.	I believe the last year led to less physical activity and binge eating.	280 (87.8%)	20 (6.3%)	2 (0.6%)	9 (2.8%)	8 (2.5%)
2.	I believe that increase in screen time has increased eye strain.	291 (91.2%)	15 (4.7%)	3 (0.9%)	6 (1.9%)	4 (1.3%)
3.	I believe that personal loss during the pandemic or the ill health of a family member has impacted my physical and mental health.	148 (46.4%)	69 (21.6%)	2 (0.6%)	63 (19.7%)	37 (11.6%)
4.	I believe that I have become more irritable over time.	139 (43.6%)	88 (27.6%)	30 (9.4%)	28 (8.8%)	34 (10.7%)
5.	I believe that my sleep has been disturbed over time.	99 (31.0%)	147 (46.1%)	29 (9.1%)	19 (6.0%)	25 (7.8%)
6.	I have experienced a lack of concentration over time.	255 (79.9%)	43 (13.5%)	10 (3.1%)	6 (1.9%)	5 (1.6%)
7.	I have experienced easy fatigue/lethargy over time.	239 (74.9%)	64 (20.1%)	1 (0.3%)	8 (2.5%)	7 (2.2%)

## Discussion

The pandemic has fundamentally changed education at every level and significantly disrupted medical education. We designed this study of 319 students to investigate the effect of COVID-19 on the academic and psychological health of medical students. The process of medical education is based on patient interaction, and the switch to online education due to unavoidable circumstances has been quite disruptive for future doctors. A 2020 survey by Harries AJ et al. of 741 medical students from six medical schools in the United States found that 74.7% agreed that the pandemic has significantly disrupted medical education. Our study found that 34.5% of participants strongly agreed, and 54.5% somewhat agreed that their education had been disrupted. The difference in findings is explainable by the difference in the number of participants in these studies [5,7]. Syal A et al., in a study at Government Medical College, Chandigarh, interviewed 196 students and reported that 46.42% felt the suspension of face-to-face, interactive classes hampered their interest in pursuing medicine. In our study, 28.2% of students felt the same. The lack of not only in-person classes but also interaction with patients, hospital visits for postings, and exposure to the work atmosphere all led to a lesser understanding of the subjects [8,9]. A 2020 survey of 164 participants with a median age of 21 years by TMS Collaborative found that 81.4% reported COVID-19 had a negative impact on their medical training [10].

Also, in 2020, a cross-sectional survey of 3,348 medical students from Libya by Alsoufi A et al. found that 47.5% reported they were good at or proficient in using electronic devices, which is similar to the results of our study. Most of the participants had good internet service, so there was no interruption to their learning experience [2]. Medical students faced several challenges. In our study, about 88% of them reported a change in their behavior personally or with family and friends, primarily due to their anxiety about contracting the disease. The results showed that 71% of them had anxiety traits, and 11% showed depressive symptoms, which may have already been present. A previous study in Libya found that 31.3% experienced depressive symptoms and 10.5% had anxiety symptoms. The pandemic was a time of great personal agony for students studying to be the doctors of the future [11,12]. Essadek A et al. conducted a comparative study in France of 8,004 students in which 668 medical students responded, and 7,336 responses were from non-medical students. Of the 668 medical students, 38.17% had depressive symptoms, 38.77% had anxiety symptoms, and 36.83% had distress symptoms. The results of our study were consistent with these studies [13]. Saraswathi I et al. surveyed 217 undergraduate medical students at a medical college in Chennai, India, to compare their anxiety and stress levels before and during the COVID-19 outbreak. The study found poor sleep quality to be a significant independent predictor of depression and anxiety during COVID-19, and our study had similar results, with 77.1% of participants reporting poor sleep quality [14]. Medical students usually have poor sleeping patterns because of their intense, mentally, and physically exhausting work. Due to lockdowns and travel restrictions, students faced decreased physical activity, a lack of schedule, altered living conditions, increased screen time leading to eye strain, and an altered sleep-wake pattern, including increased daytime nap duration. In this study, we found a significant association between testing for COVID-19 and anxiety and stress because most of the students were infected during the second wave of COVID-19 in India [15].

## Conclusions

This study has shed light on the effect of COVID-19 contagion on MBBS scholars. In accordance with Sustainable Development Goal 3, which focuses on "good health and well-being, this study shows how students have responded to this unparalleled situation. The knowledge gained from this study will assist facilitators and students of the medical fraternity in carrying out effective teaching modalities during the pandemic and any future outbreaks.
